# Robotic versus open surgery for gallbladder cancer: a meta-analysis of propensity-score-matched studies

**DOI:** 10.3389/fonc.2026.1841955

**Published:** 2026-06-10

**Authors:** Piyun Zhang, Li Zhou, Lin Wu

**Affiliations:** 1 Department of Gastroenterology, Chongqing University Central Hospital & Chongqing Emergency Medical Center, Chongqing University, Chongqing, China; 2 Department of Hepatobiliary, Chongqing University Central Hospital & Chongqing Emergency Medical Center, Chongqing University, Chongqing, China

**Keywords:** gallbladder cancer, meta-analysis, morbidity, open radical cholecystectomy, robotic radical cholecystectomy

## Abstract

**Background:**

The use of robotic surgery for gallbladder cancer (GBC) has increased in recent years. However, concerns remain regarding the safety and oncologic efficacy of robotic-assisted surgery for GBC.

**Objective:**

This systematic review and meta-analysis aimed to compare the safety and efficacy of robotic radical cholecystectomy (RRC) versus open radical cholecystectomy (ORC).

**Methods:**

The PubMed, Cochrane Library, Scopus, EMBASE, and Web of Science databases were searched to identify available research published up to January 13, 2026. Odds ratios (ORs) and mean differences (MDs) with 95% confidence intervals (CIs) were calculated.

**Results:**

A total of 5 propensity-score-matched studies including 629 participants (RRC group: 202 patients; ORC group: 427 patients) were included. Compared with the ORC group, the RRC group had fewer major complications (OR, 0.42), lower VAS scores (MD, −0.53), less intraoperative blood loss (MD, −114.77 mL), and shorter hospital stay (MD, −2.92 days). No significant differences were observed between the groups in mortality (OR, 1.38), overall morbidity (OR, 0.75), operative time (MD, 2.11 min), number of harvested lymph nodes (MD, 0.13), R0 resection (OR, 1.14), blood transfusion (OR, 0.25), readmission (OR, 1.22), 3-year survival rate (OR, 1.01), 5-year survival rate (OR, 1.04), 3-year disease-free survival (OR, 1.02), or 5-year disease-free survival (OR, 1.13).

**Conclusions:**

This meta-analysis suggests that robotic surgery is a safe and effective approach for the surgical management of GBC, with postoperative and survival outcomes comparable to those of open surgery. Additionally, robotic surgery may be associated with shorter hospital stay, fewer major complications, reduced intraoperative blood loss, and lower VAS scores compared with open surgery. Further randomized controlled trials are warranted to confirm the potential advantages of RRC over ORC.

**Systematic review registration:**

https://www.crd.york.ac.uk/PROSPERO/, identifier CRD420261364335.

## 1 Introduction

Gallbladder cancer (GBC) is the most common malignancy of the biliary tract and ranks sixth among gastrointestinal cancers in incidence (1). Although relatively rare, GBC is highly aggressive and is associated with a poor prognosis (2). Radical resection remains the only potentially curative treatment for this disease (2, 3). Over the past several decades, open surgery has been regarded as the standard surgical approach for GBC. Despite its proven efficacy, traditional open radical cholecystectomy is associated with substantial postoperative morbidity, which may adversely affect patient recovery (3, 4).

Compared with conventional open surgery, laparoscopic approaches offer several advantages, including reduced intraoperative blood loss, lower rates of postoperative complications, and faster recovery, and have been widely adopted in hepatobiliary and pancreatic surgery (5–7). Several studies have demonstrated that laparoscopic radical cholecystectomy achieves perioperative and long-term survival outcomes comparable to those of open surgery (8–10). However, laparoscopic surgery has inherent limitations, including a two-dimensional visual field that compromises depth perception, restricted instrument dexterity, and unavoidable physiological tremor (4).

Robotic surgical platforms have been developed to overcome these limitations while preserving the benefits of minimally invasive surgery (11, 12). Several studies have compared robotic and open approaches for radical resection of GBC in terms of perioperative outcomes; however, the results remain inconclusive (13–16). Goel et al. (13) reported that, compared with open surgery, robotic surgery was associated with significantly reduced intraoperative blood loss, shorter hospital stay, and lower postoperative complication rates. In contrast, Wållgren et al. (14) found no significant difference in postoperative complications between robotic and open approaches. Nevertheless, the majority of existing evidence is derived from retrospective studies, which inherently provide a lower level of evidence. Furthermore, imbalances in baseline characteristics between comparison groups may compromise the validity of these findings. Propensity score matching (PSM) is an effective statistical approach to minimize such confounding by balancing baseline covariates between groups (17). Notably, well-designed PSM studies have been shown to approximate the methodological rigor of randomized controlled trials (RCTs) (18). In recent years, several high-quality PSM studies (19–23) on this topic have been published; however, a comprehensive meta-analysis based on these studies is still lacking.

Therefore, in the present study, we conducted a meta-analysis based on PSM studies to compare the surgical outcomes between robotic and open approaches for the treatment of GBC. Our findings aim to provide high-quality evidence to inform surgical decision-making and optimize the selection of operative approaches for patients with gallbladder cancer.

## 2 Methods

### 2.1 Search strategy

This meta-analysis was conducted in accordance with the Preferred Reporting Items for Systematic Reviews and Meta-Analyses (PRISMA) guidelines. The study was registered in the PROSPERO database (registration number: CRD420261364335).

PubMed, Cochrane Library, Scopus, EMBASE, and Web of Science were systematically searched from inception to January 13, 2026. Search terms are presented in **Table 1**. Two authors (P.Z. and L.Z.) independently performed the literature search. In addition, we checked the reference lists of the identified articles and related reviews to further screen for eligible studies. No time or language restrictions were applied during the search process.

**Table 1:** Electronic search strategy.

Database	Search term (published up to January 13, 2026)	Number
PubMed (all fields)	((Robot*) OR (Robot-assisted) OR (Robotic-assisted) OR (Da Vinci) OR Robotic) AND ((gallbladder carcinoma) OR (gallbladder cancer))	109
Embase (all fields)	(gallbladder carcinoma OR gallbladder cancer) AND (Da Vinci OR Robot* OR Robot-assisted OR Robotic-assisted OR Robotic)	331
Cochrane library trials (all fields)	((Da Vinci) OR Robot* OR Robot-assisted OR Robotic-assisted OR Robotic) AND (gallbladder carcinoma OR gallbladder cancer)	7
Web of science (topic)	(TS=((Da Vinci) OR (Robot*) OR (Robot-assisted) OR (Robotic-assisted) OR Robotic)) AND TS=((gallbladder carcinoma) OR (gallbladder cancer))	138
Scopus (title, abstract, keywords)	((Da Vinci) OR Robot* OR Robot-assisted OR Robotic-assisted OR Robotic) AND (gallbladder carcinoma OR gallbladder cancer)	159

### 2.2 Study selection

Inclusion criteria were as follows:

1. Population: patients diagnosed with gallbladder cancer;
2. Intervention: robotic radical cholecystectomy (RRC);
3. Comparison: open radical cholecystectomy (ORC);
4. Outcomes: primary outcomes included mortality, overall morbidity, major complications (Clavien–Dindo ≥3), and length of stay. Secondary outcomes included blood loss, operative time, readmission, blood transfusion, visual analog scale (VAS) score, number of lymph nodes harvested, R0 resection, and survival outcomes (3-year survival rate, 5-year survival rate, 3-year disease-free survival [DFS], and 5-year DFS);
5. Study design: Randomized controlled trials (RCTs) were sought but none were identified; only PSM studies were included.
  Non-PSM studies, laboratory studies, repeated publications, reviews, letters, case reports, and conference abstracts were excluded.

### 2.3 Data extraction

Data from each eligible study were independently extracted by two authors (P.Z. and L.Z.) using a standardized data collection form, and any discrepancies were resolved through consultation with a third author (L.W.). Extracted data included author name, year of publication, country, study design, study population (sample size, age, and sex), and outcomes (mortality, overall morbidity, major complications, length of stay, blood loss, operative time, readmission, blood transfusion, visual analog scale (VAS) score, number of lymph nodes harvested, R0 resection and survival outcomes).

### 2.4 Quality assessment

The quality assessment was conducted independently by two authors according to the Newcastle-Ottawa Scale (NOS). A score greater than 6 was considered indicative of high quality. Any disagreements were resolved through discussion, with arbitration by a third author when necessary.

### 2.5 Statistical analysis

Review Manager (RevMan, version 5.3) was used for this systematic review and meta-analysis. For continuous outcomes, mean differences (MDs) with corresponding 95% confidence intervals (CIs) were calculated, whereas odds ratios (ORs) with 95% CIs were used for dichotomous outcomes. The inconsistency index (I²) was used to quantify the degree of statistical heterogeneity. If significant heterogeneity was detected (I²> 50%), a random-effects model was applied; otherwise, a fixed-effects model was used (24). To assess the robustness of the results, a leave-one-out sensitivity analysis was performed to evaluate the influence of each individual study on the overall effect size. In addition, supplementary sensitivity analyses using a random-effects model were conducted to further evaluate the stability of the pooled estimates. Potential publication bias was evaluated using funnel plots and Egger’s test. A P value <0.05 was considered statistically significant.

## 3 Results

### 3.1 Study selection

**Figure 1:**
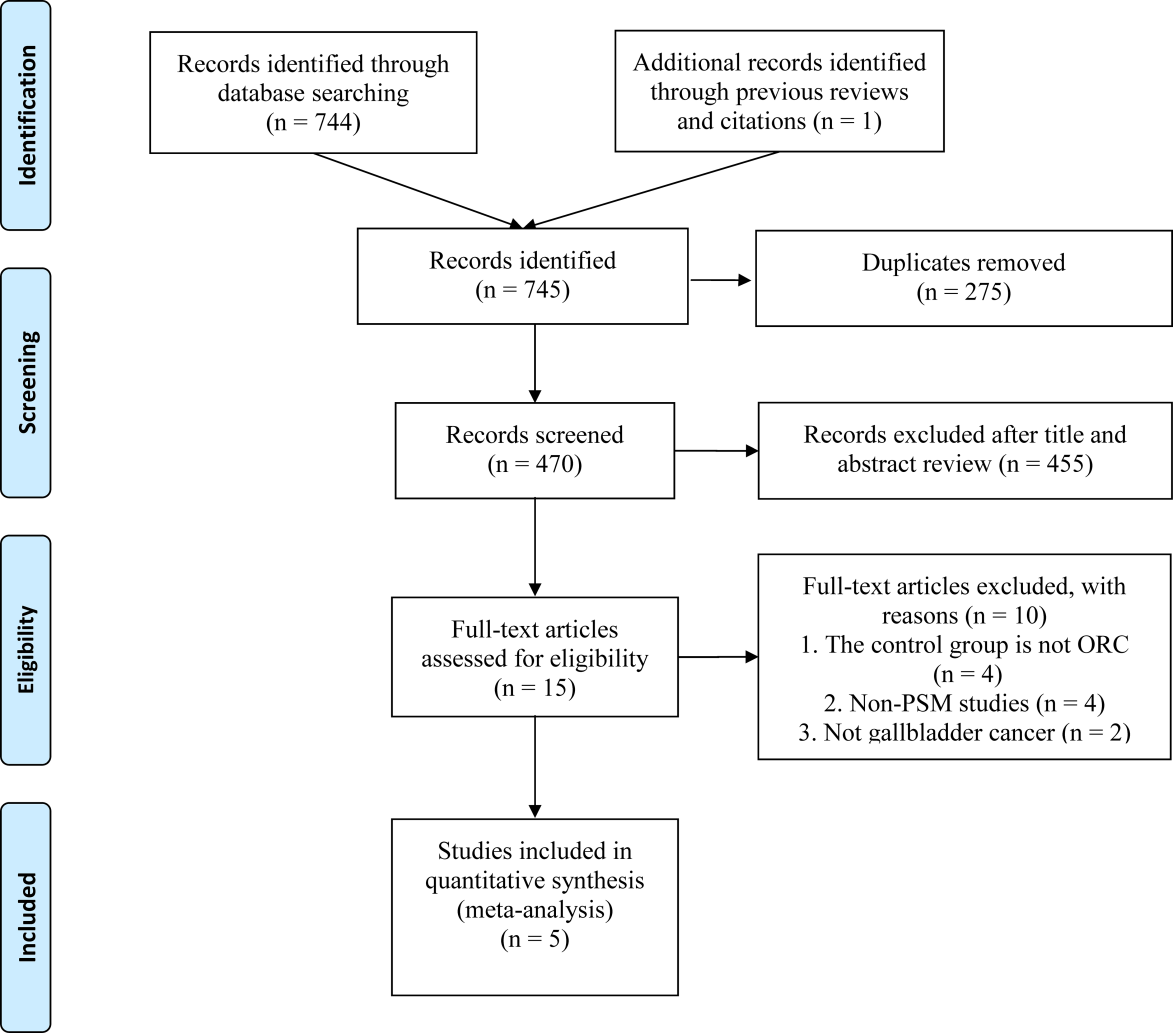
The PRISMA flowchart.

A total of 745 studies were identified from five databases, of which 275 duplicates were removed. Following title and abstract screening, 455 studies were excluded, and the full texts of the remaining 15 studies were assessed for eligibility. Ultimately, 5 studies (19–23) met the inclusion criteria and were included in the final analysis (**Figure 1**).

### 3.2 Study characteristics and quality assessment

The detailed characteristics of the included studies (19–23) are summarized in **Table 2**. These studies were conducted in various countries, including the South Korea, China, and Italy, and all were cohort studies. The studies were published between 2020 and 2024, encompassing a total of 629 patients (RRC group: 202; ORC group: 427). All included studies (19–23) were considered high quality, with NOS scores greater than 6.

**Table 2:** Study characteristics of the 5 included studies.

First author, year	Country	Period of study	Male	Study type	Age	Sample size	Follow-up (months), mean ± SD or mean/median (range)	NOS
Byun 2020	Korea	2018-2019	RRC:8 ORC: 22	RCS, PSM	RRC: 63.5(10.5) ORC: 65(10.5)	RRC:13 ORC: 39	RRC: NA ORC: NA	8/9
Yang 2022	China	2015-2022	RRC: 16 ORC: 29	RCS, PSM	RRC: 58.5 (12.15) ORC: 57.08 (13.82)	RRC:28 ORC: 51	RRC: 16 (10.7) ORC: 20.1 (12.6)	8/9
Cho 2023	Korea	2018-2021	RRC: 25 ORC: 24	RCS, PSM	RRC: 66.8 (9) ORC: 68.8 (8)	RRC: 41 ORC: 41	RRC: NA ORC: NA	8/9
Ielpo 2024	Italy	2012-2022	RRC:35 ORC: 32	RCS, PSM	RRC: 67.83 (11.3) ORC: 66.17 (11.96)	RRC: 98 ORC: 98	RRC: 42.7 (11-96) ORC: 42.7 (11-96)	9/9
Sohn 2024	Korea	2010-2020	RRC: 9 ORC: 116	RCS, PSM	RRC: 67.6 (9.5) ORC: 67.2 (9.3)	RRC: 22 ORC: 198	RRC: NA ORC: NA	8/9

NA, not available; ORC: open radical cholecystectomy; PSM, propensity score matching; RCS, retrospective cohort study; RRC, robotic radical cholecystectomy.

### 3.3 Meta-analysis

#### 3.3.1 Mortality

**Figure 2:**
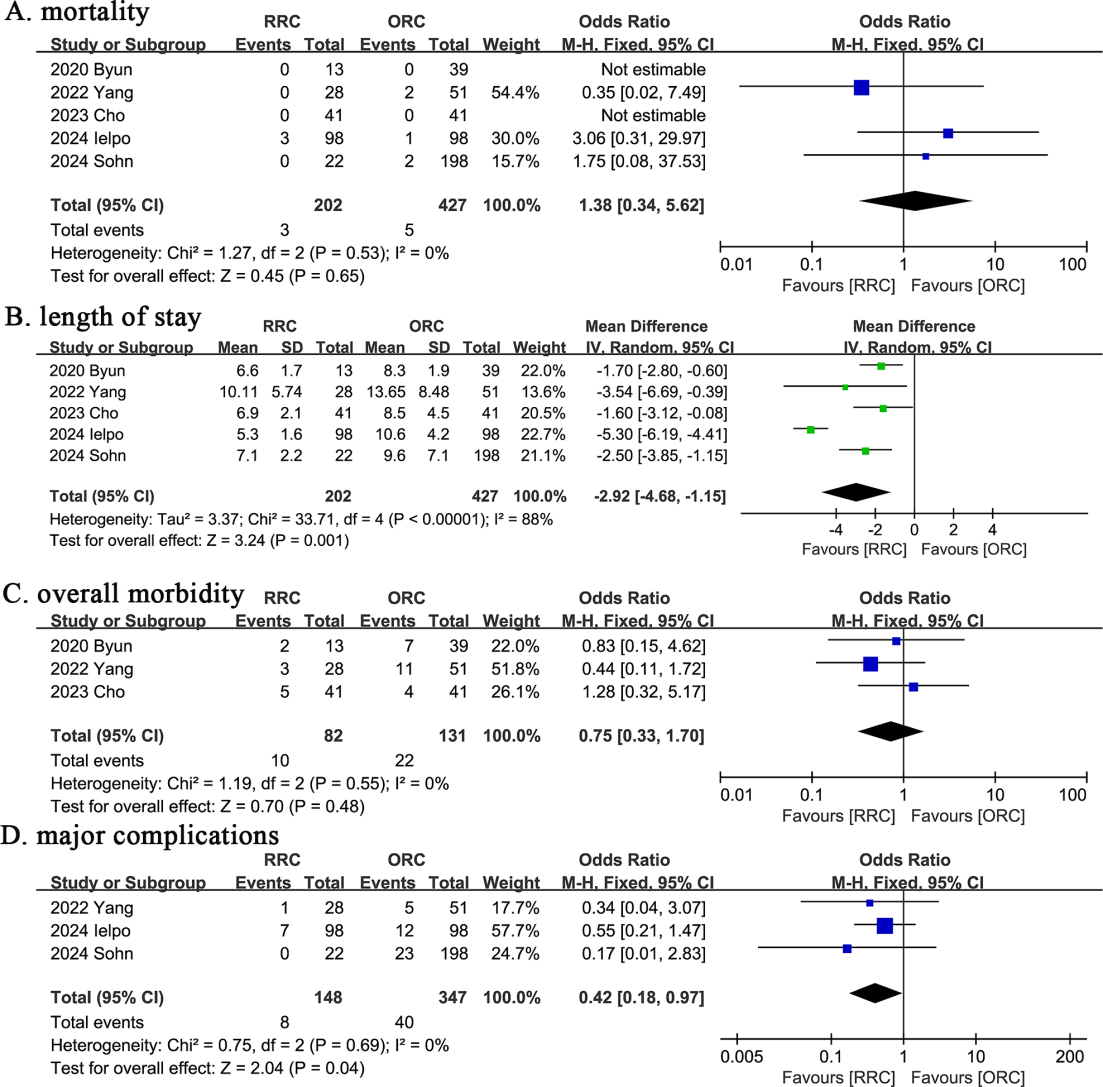
Comparison of primary outcomes between the two groups. **(A)** mortality, **(B)** length of stay, **(C)** overall morbidity, and **(D)** major complications.

Five studies (19–23) assessed mortality. The combined results of the 5 studies showed that there was no significant difference between the RRC group and the ORC group regarding this outcome with low heterogeneity (OR 1.38, 95% CI 0.34, 5.62; Heterogeneity: I^2^ = 0%, P = 0.53) (**Figure 2A****, ****Table 3****).**

**Table 3:** Summary of results from all outcomes.

Outcomes	No. of studies	Events for RRC	Events for ORC	Effect size	95%CI	P	I^2^ (%)
Overall complications	3	10/82	22/131	0.75	0.33, 1.70	0.48	0
Mortality	5	3/202	5/427	1.38	0.34, 5.62	0.65	0
Major complications	3	8/148	40/347	0.42	0.18, 0.97	0.04	0
Blood transfusion	2	1/63	14/239	0.25	0.04, 1.50	0.13	0
R0 resection	3	145/161	307/337	1.14	0.56, 2.33	0.72	0
Readmission	2	12/139	10/139	1.22	0.51, 2.94	0.66	0
3-year survival rate	3	117/167	133/190	1.01	0.62, 1.63	0.97	24
5-year survival rate	2	85/139	84/139	1.04	0.61, 1.78	0.89	10
3-year DFS	3	95/167	106/190	1.02	0.66, 1.59	0.92	13
5-year DFS	2	77/139	73/139	1.13	0.69, 1.85	0.62	0
VAS score	2	–	–	-0.53	-1.01, -0.04	0.03	0
Blood loss	5	–	–	-114.77	-148.89, -80.66	< 0.00001	46
Operative time	5	–	–	2.11	-7.34, 11.57	0.66	30
Number of lymph nodes harvested	5	–	–	0.13	-1.32, 1.58	0.86	82
Hospital stay	5	–	–	-2.92	-4.68, -1.15	0.001	88

#### 3.3.2 Length of stay

Length of the hospital stay was reported in 5 studies (19–23). According to the results of this meta-analysis, RRC significantly reduced the length of hospital stay (MD, -2.92 days; 95% CI, -4.68, -1.15, P = 0.001) (**Figure 2B**).

#### 3.3.3 Morbidity

Three studies (19–21) reported data on overall complication. The pooled results suggested that RRC did not significantly reduce the overall complication rate (OR 0.75, 95% CI 0.33, 1.70, P = 0.48), with low heterogeneity (I^2^ = 0%, P = 0.55) (**Figure 2C**). Pooled data from three studies (20, 22, 23) demonstrated that, compared with ORC, RRC was associated with a significantly lower incidence of major complications (Clavien–Dindo ≥ 3) (OR 0.42, 95% CI 0.18, 0.97; Heterogeneity: I^2^ = 0%, P = 0.69) (**Figure 2D**).

#### 3.3.4 Blood loss

**Figure 3:**
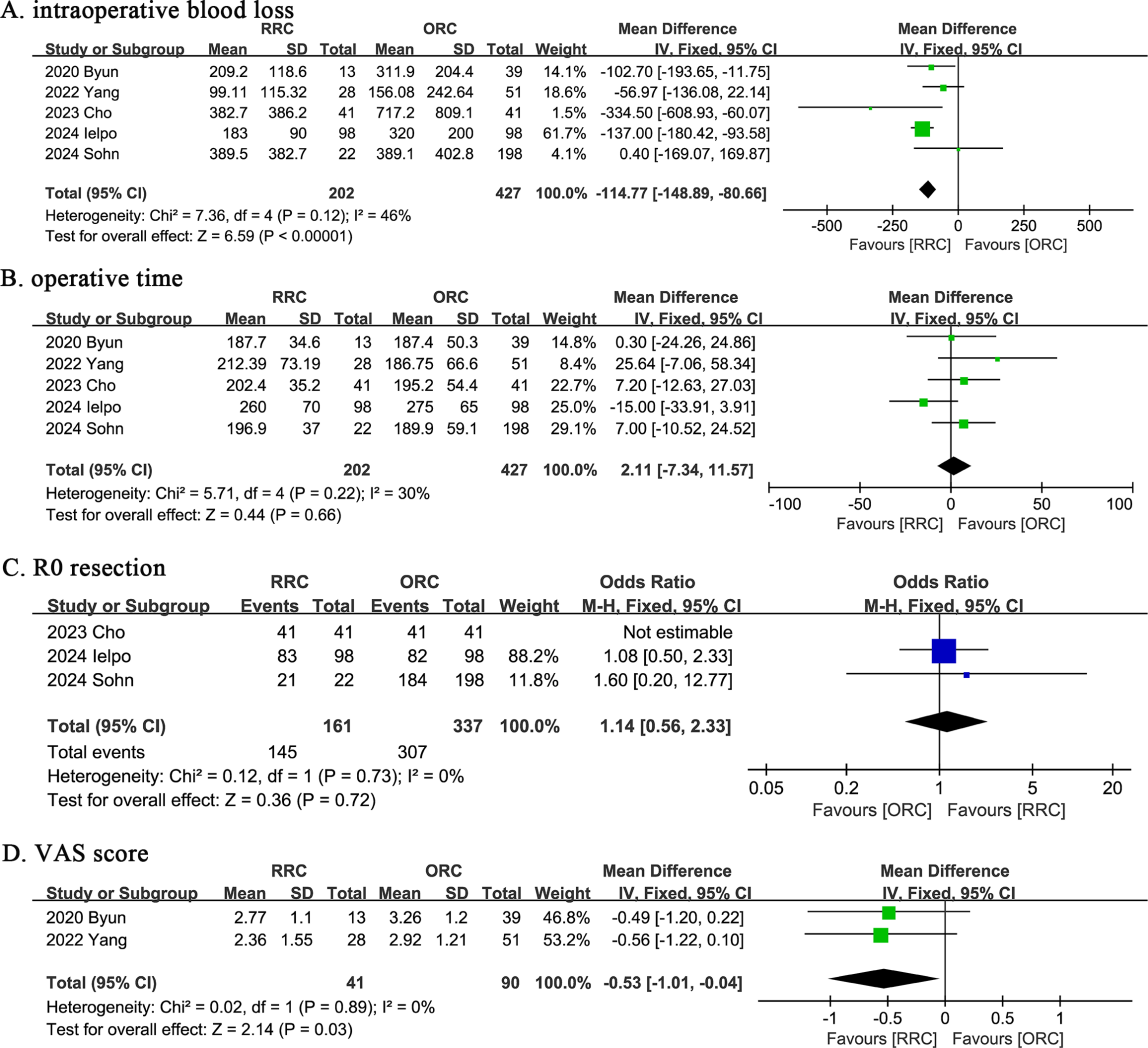
Comparison of secondary outcomes between the two groups. **(A)** intraoperative blood loss, **(B)** operative time, **(C)** R0 resection, and **(D)** VAS score.

The intraoperative blood loss was reported in 5 studies (19–23). The pooled analysis showed that the RRC group had significantly less intraoperative blood loss than the ORC group (MD, -114.77 mL; 95% CI, -148.89, -80.66, P < 0.00001) (**Figure 3A**).

#### 3.3.5 Operative time

Five studies (19–23) provided information on operative time. The combined results showed that the operative time was similar between the RRC group and the ORC group (MD, 2.11 min; 95% CI, -7.34, 11.57, P = 0.66; I^2^ = 30%) (**Figure 3B**).

#### 3.3.6 R0 resection

R0 resection was reported in 3 studies (21–23), and the combined effect size suggested that the R0 resection rates were comparable between the two groups (OR 1.14, 95% CI 0.56, 2.33, P = 0.72; I^2^ = 0%) (**Figure 3C**).

#### 3.3.7 VAS score

Two studies (19, 20) provided information on VAS score. The pooled analysis demonstrated that VAS score was significantly lower in the RRC group than in the ORC group (MD, -0.53; 95% CI, -1.01, -0.04, P = 0.03) (**Figure 3D**).

#### 3.3.8 Number of lymph nodes harvested

**Figure 4:**
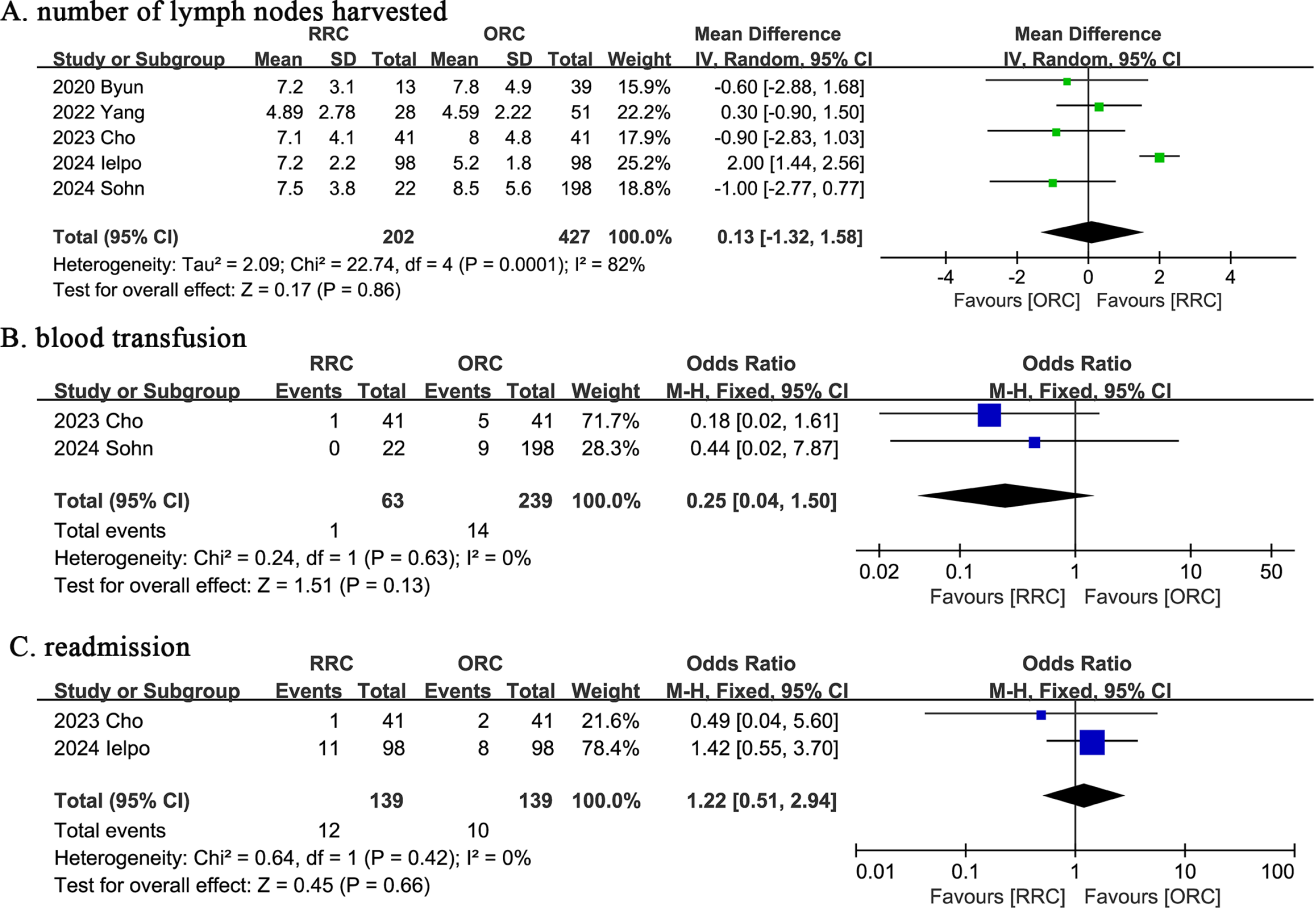
Comparison of secondary outcomes between the two groups. **(A)** number of lymph nodes harvested, **(B)** blood transfusion, and **(C)** readmission.

Five trials (19–23) reported the number of lymph nodes harvested. There was no significant difference in the number of lymph nodes harvested (MD, 0.13; 95% CI, -1.32, 1.58, P = 0.86; I^2^ = 82%) (**Figure 4A**).

#### 3.3.9 Blood transfusion

Two studies (21, 23) compared blood transfusion rates between the RRC and ORC groups. There was no significant difference in blood transfusion rate (OR 0.25, 95% CI 0.04, 1.50, P = 0.13) (**Figure 4B**).

#### 3.3.10 Readmission

Two trials (21, 22) reported the readmission rates. There were no significant differences between the two groups, and heterogeneity was low (OR 1.22, 95% CI 0.51, 2.94; Heterogeneity: I^2^ = 0%, P = 0.42; **Figure 4C**).

#### 3.3.11 3-year survival rate

**Figure 5:**
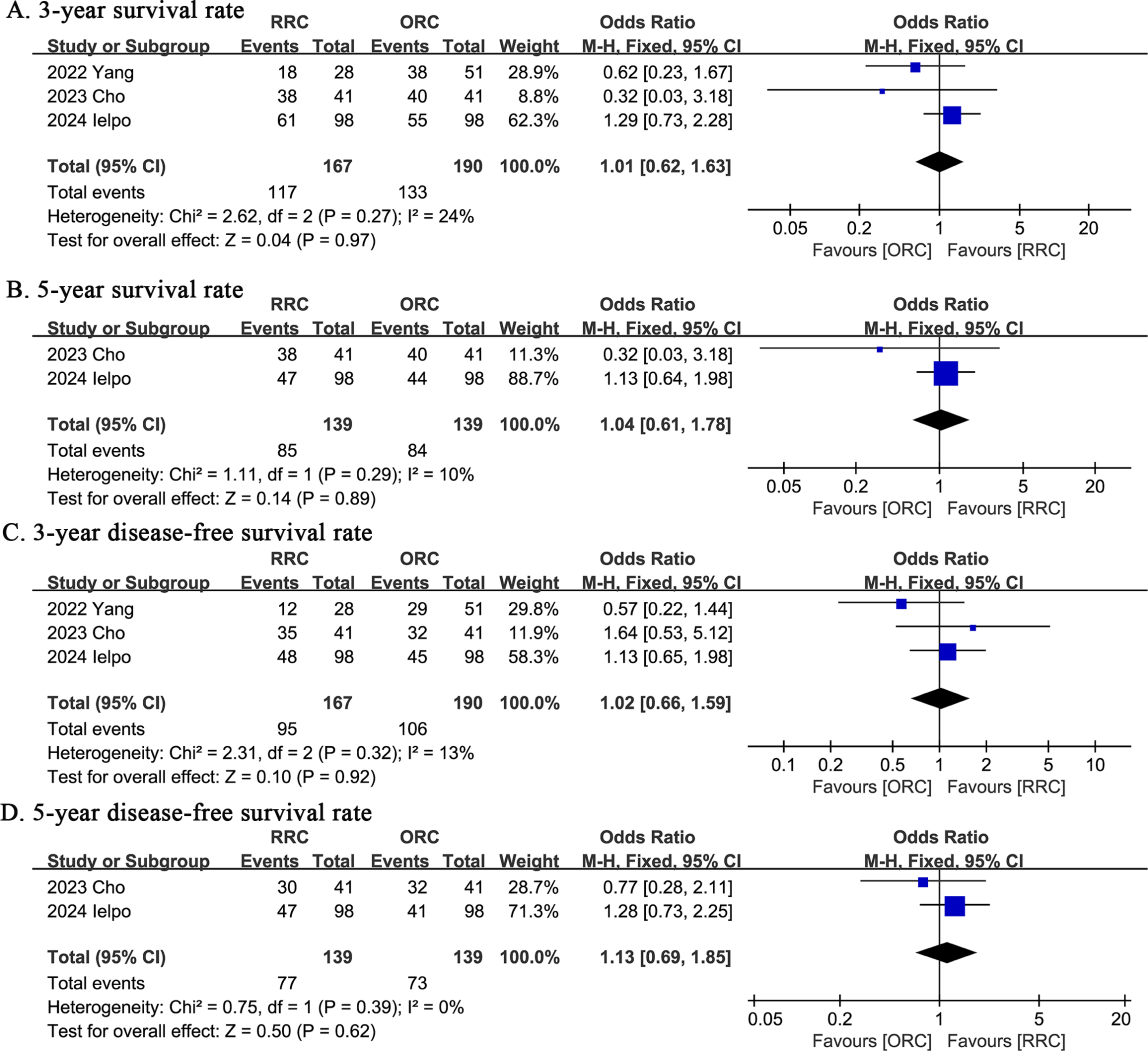
Comparison of secondary outcomes between the two groups. **(A)** 3-year survival rate, **(B)** 5-year survival rate, **(C)** 3-year disease-free survival rate, and **(D)** 5-year disease-free survival rate.

Three studies (20–22) evaluated the 3-year survival rate. There was no significant difference in the 3-year survival rate (OR 1.01, 95% CI 0.62, 1.63, P = 0.97) (**Figure 5A**) between the RRC and ORC groups.

#### 3.3.12 5-year survival rate

Two studies (21, 22) reported the 5-year survival rate. No significant differences were observed between the two groups (OR 1.04, 95% CI 0.61, 1.78, P = 0.89), and heterogeneity was low (I^2^ = 10%, P = 0.29) (**Figure 5B**).

#### 3.3.13 3-year disease-free survival rate

3-year DFS was reported in 3 studies (20–22), and the combined effect size suggested that the 3-year DFS was comparable between the two groups (OR 1.02, 95% CI 0.66, 1.59, P = 0.92; I^2^ = 13%) (**Figure 5C**).

#### 3.3.14 5-year disease-free survival rate

5-year DFS was evaluated in 2 studies (21, 22), and the pooled analysis demonstrated no significant difference in 5-year DFS between the two groups (OR 1.13, 95% CI 0.69, 1.85; heterogeneity: I^2^ = 0%, P = 0.39) (**Figure 5D**).

### 3.4 Publication bias and sensitivity analysis

**Figure 6:**
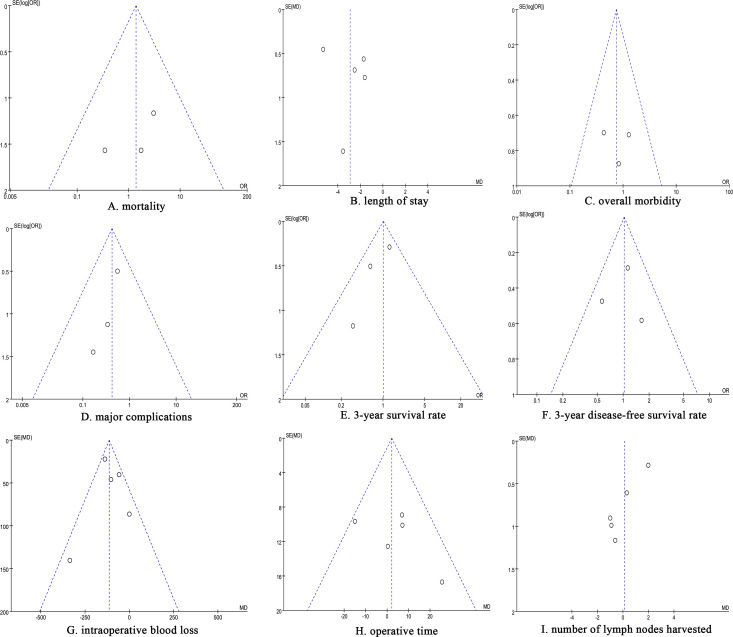
Funnel plot of primary outcomes. **(A)** mortality, **(B)** length of stay, **(C)** overall morbidity, **(D)** major complications, **(E)** 3-year survival rate, **(F)** 3-year disease-free survival rate, **(G)** intraoperative blood loss, **(H)** operative time, and **(I)** number of lymph nodes harvested.Tables.

According to funnel plots and Egger’s test (**Figure 6**), no significant publication bias was observed for mortality (P = 0.449), overall morbidity (P = 0.858), major complications (P = 0.858), length of stay (P = 0.140), 3-year survival rate (P = 0.255), 3-year disease-free survival rate (P = 0.935), intraoperative blood loss (P = 0.816), operative time (P = 0.388), and number of lymph nodes harvested (P = 0.06). Leave-one-out sensitivity analysis demonstrated that no individual study substantially influenced the pooled effect estimates for mortality, overall morbidity, length of hospital stay, intraoperative blood loss, operative time, number of harvested lymph nodes, R0 resection rate, 3-year overall survival, or 3-year disease-free survival. The sensitivity analysis suggested that the total effect size of major complications changed significantly when the study by Yang et al. (20) (OR 0.44, 95% CI 0.18, 1.08; I^2^ = 0%, P = 0.41), Ielpo et al. (22) (OR 0.24, 95% CI 0.04, 1.35; I^2^ = 0%, P = 0.69) or the study by Sohn et al. (23) (OR 0.50, 95% CI 0.21, 1.22; I^2^ = 0%, P = 0.69) was excluded (**Supplementary Figure 1**). Additional sensitivity analyses using a random-effects model demonstrated that the pooled results for mortality, overall morbidity, length of hospital stay, intraoperative blood loss, operative time, number of harvested lymph nodes, R0 resection rate, VAS score, blood transfusion, readmission, 3-year overall survival, 3-year disease-free survival, 5-year overall survival, and 5-year disease-free survival remained materially unchanged. However, when the effect model for major complications was switched to a random-effects model, the pooled effect estimate changed substantially (OR 0.46, 95% CI 0.20, 1.08; I^2^ = 0%, P = 0.69) (**Supplementary Figure 1**).

## 4 Discussion

In recent years, robotic surgery has attracted considerable attention in hepatobiliary and pancreatic surgery. However, its application in gallbladder cancer (GBC) remains limited due to the lack of compelling evidence supporting its clinical value in radical resection. Based on pooled data from five PSM studies, our meta-analysis demonstrated that RRC was associated with a significantly lower incidence of major postoperative complications, shorter hospital stay, reduced intraoperative blood loss, and lower postoperative VAS score. Meanwhile, no significant differences were observed between RRC and ORC in terms of overall complications, mortality, operative time, R0 resection rate, or lymph node yield. In addition, long-term outcomes, including 3-year and 5-year OS and DFS, were comparable between the two approaches.

The robotic surgical platform offers several technical advantages, including magnified visualization, enhanced dexterity with articulating instruments, and elimination of physiological tremor, which may contribute to improved perioperative outcomes in complex abdominal oncologic procedures (23, 25–27). Chen et al. (28) reported that robotic gastrectomy was associated with reduced intraoperative blood loss, shorter hospital stay, and fewer postoperative complications compared with open surgery. Similarly, Luo et al. (25) demonstrated that robot-assisted cystectomy significantly reduced blood loss, length of stay, and positive surgical margin rates. Furthermore, a recent meta-analysis by Waseem et al. (26) indicated that even in highly complex procedures such as pancreaticoduodenectomy, robotic surgery was associated with lower postoperative complication rates and shorter hospitalization. Postoperative complications not only prolong hospitalization and increase healthcare costs but also adversely affect long-term outcomes (29). Notably, the incidence of postoperative complications following open radical resection for GBC has been reported to range from 13% to 57% (10, 30). The meta-analysis by Wang et al. indicated that the overall postoperative complication rate of robotic radical cholecystectomy is significantly lower than that of the open surgery group (3). Consistent with previous findings, our analysis showed that the rates of overall and major complications were 16.8% and 11.5% in the ORC group, compared with 12.2% and 5.4% in the RRC group, respectively. Although no significant difference was observed in overall complication rates, robotic surgery significantly reduced the incidence of major complications. Nevertheless, given the limited number of included studies, further research is warranted to validate these findings.

The minimally invasive nature of robotic surgery contributes to reduced surgical trauma and, consequently, lower intraoperative blood loss. In addition, the high-definition three-dimensional visualization provided by robotic systems facilitates precise dissection and hemostasis (3, 26). Waseem et al. reported reduced blood loss in robotic pancreaticoduodenectomy (26). In our study, pooled results demonstrated significantly lower intraoperative blood loss in the robotic group compared with the open group. This finding is further supported by a retrospective study by Ratti et al. (31) involving 138 patients undergoing biliary tumor resection, which showed a significant reduction in blood loss with robotic surgery compared with open surgery (median 200 mL vs. 350 mL, P = 0.03). These results are consistent with our findings and further support the advantage of robotic surgery in minimizing intraoperative blood loss. Additionally, although a lower transfusion rate was observed in the robotic group, the difference did not reach statistical significance.

Lymph node dissection and R0 resection rate are critical indicators of oncologic adequacy in GBC surgery. Previous studies have reported a lymph node metastasis rate of up to 46% in patients with T2-stage GBC (32, 33). Adequate lymph node retrieval is closely associated with accurate staging and improved long-term outcomes, with at least 6 lymph nodes generally recommended for pathological evaluation (19, 21, 33). It has been suggested that robotic approaches may offer advantages in lymphadenectomy due to superior visualization and instrument flexibility (19, 20). However, in our study, the mean number of retrieved lymph nodes was comparable between the robotic and open groups (6.7 vs. 6.8), with no significant difference observed. For resectable gallbladder adenocarcinoma, achieving R0 resection is strongly associated with improved overall survival (34). Our analysis demonstrated no significant difference in R0 resection rates between the two approaches. Furthermore, long-term survival outcomes, including 3-year and 5-year OS and DFS, were also comparable, further supporting the oncologic adequacy of the robotic approach.

The larger incision required for open surgery is often associated with prolonged recovery and extended hospital stay (3). In contrast, minimally invasive approaches are theoretically associated with reduced surgical stress, earlier ambulation, and faster recovery of gastrointestinal function, thereby shortening hospitalization (30). These advantages have been confirmed in previous meta-analyses (11, 35, 36). Huang et al. (11) reported that robotic surgery significantly shortened time to first flatus, time to ambulation, and length of hospital stay. Moreover, the smaller incisions associated with robotic surgery contribute to reduced postoperative pain (3). Consistent with these findings, our study demonstrated that robotic surgery significantly reduced both hospital stay and postoperative pain scores. Notably, standard operative procedures may vary across centers, including differences in port placement, incision design, specimen extraction sites, and other technical details. These factors are known to influence postoperative pain and recovery, which may consequently contribute to variability in postoperative outcomes, particularly VAS scores and recovery-related indicators. However, most included studies did not provide sufficiently detailed or standardized information regarding operative setup, trocar configuration, extraction site selection, or postoperative analgesic protocols. Therefore, quantitative analysis of these factors was not feasible in the present study. Future studies are warranted to better control for and balance the influence of these perioperative variables.

This study has several strengths. First, we conducted a comprehensive literature search across 5 major databases (PubMed, Cochrane Library, Scopus, EMBASE, and Web of Science), thereby minimizing potential selection bias. Second, only PSM studies were included, which enhances the reliability and comparability of the results.

There are several limitations to our meta-analysis. First, due to the low incidence of GBC, most included studies had relatively small sample sizes, which may introduce potential bias. Second, all included studies were retrospective in nature, and although PSM was applied, residual confounding cannot be excluded. Therefore, well-designed prospective RCTs are needed to further validate the comparative effectiveness of robotic and open approaches. In addition, most of the studies included in the analysis were conducted in Asian populations. Therefore, the conclusions of this meta-analysis may be more applicable to Asian patients, as patient characteristics may differ substantially between Asian and non-Asian populations. Further multicenter studies involving more diverse populations are needed to validate the external generalizability of our findings. Moreover, because only five studies were included, the interpretation of funnel plots should be approached with caution, as Egger’s test has limited statistical power in meta-analyses with a small number of studies. Accordingly, the P values derived from publication bias analyses should not be overinterpreted. Furthermore, sensitivity analyses suggested that the results regarding major complications were not sufficiently robust and should therefore be interpreted cautiously until validated in larger prospective studies. Finally, despite the use of a random-effects model, substantial heterogeneity was observed in outcomes such as lymph node yield and length of hospital stay. This heterogeneity may be attributable to differences in the extent of surgery, surgeon experience, and patient characteristics across studies. However, due to the limited number of included studies, further subgroup analyses could not be performed.

In conclusion, robotic surgery appears to be a safe and effective approach for the treatment of GBC, providing surgical outcomes comparable to those of open surgery, while offering potential advantages in reducing major postoperative complications, intraoperative blood loss, postoperative pain, and length of hospital stay. Future randomized controlled trials are warranted to further establish the clinical value of robotic surgery in radical resection for gallbladder cancer.

## Data Availability

The original contributions presented in the study are included in the article/**Supplementary Material**. Further inquiries can be directed to the corresponding author.
