# Supplementary figures and images for: Robotic versus open surgery for gallbladder cancer: a meta-analysis of propensity-score-matched studies
Source: Front Oncol. 2026 Jun 10;16:1841955. doi: 10.3389/fonc.2026.1841955 (PMC13290461; doi:10.3389/fonc.2026.1841955)

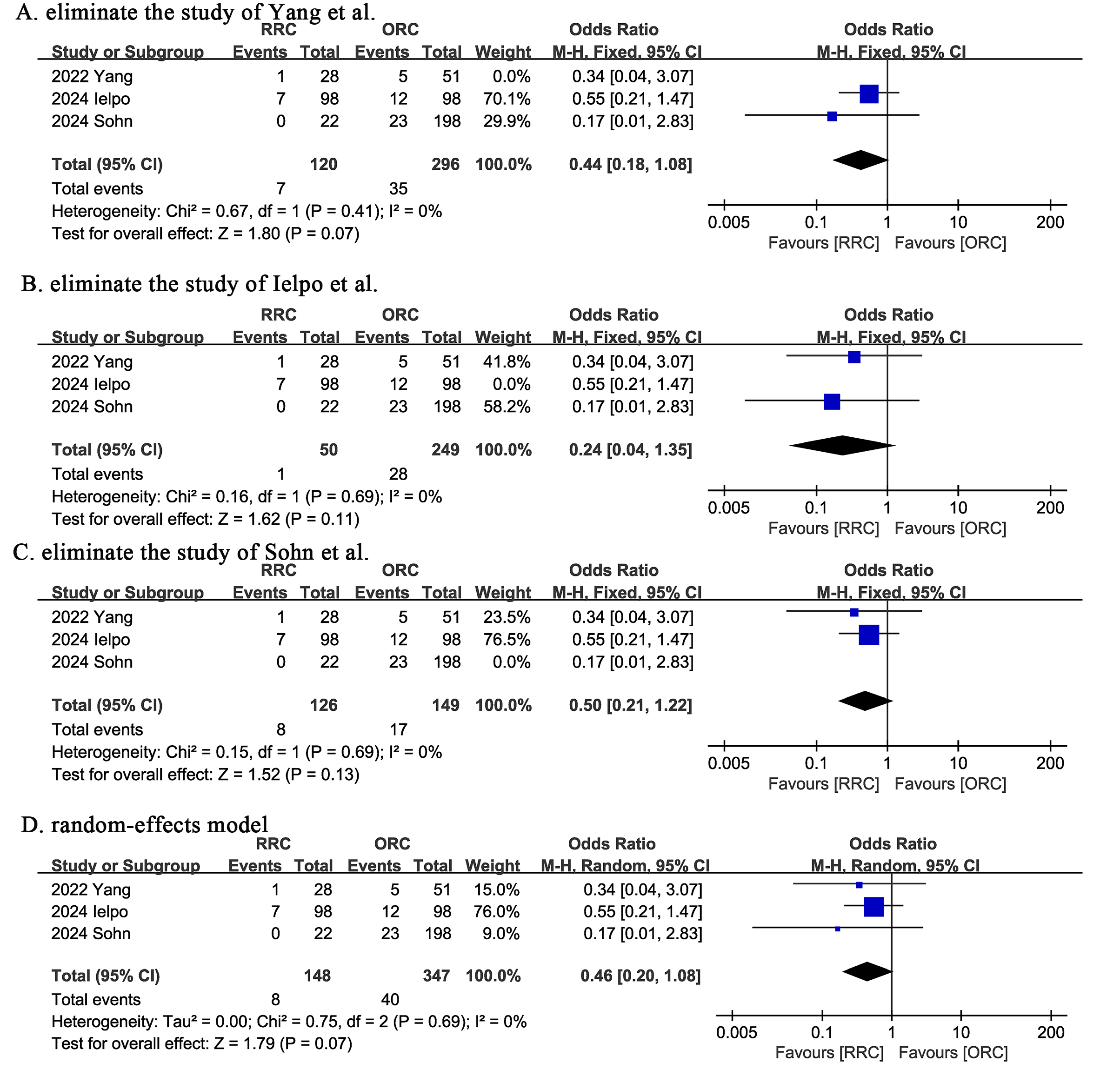

Supplement: Supplementary file 1 [file Image1.tif]
